# Breast-Conserving Surgery in *BRCA* Mutation Carriers: A Paradigm Shift Toward Individualized, Multidisciplinary Care

**DOI:** 10.3390/life15111701

**Published:** 2025-11-03

**Authors:** Calogero Cipolla, Giuseppa Scandurra, Daniela Sambataro, Chiara Mesi, Martina Greco, Eleonora D’Agati, Vittorio Gebbia, Luca Giacomelli, Maria Rosaria Valerio

**Affiliations:** 1 Breast Unit, AOUP Paolo Giaccone, 90127 Palermo, Italy; calogero.cipolla@unipa.it; 2 Department of Biomedicina, Neuroscienze e Diagnostica Avanzata, University of Palermo, 90127 Palermo, Italy; 3 Medical Oncology, Department of Medicine and Surgery, Kore University of Enna, 94019 Enna, Italy; giuseppa.scandurra@unikore.it (G.S.); daniela.sambataro@unikore.it (D.S.); 4 Medical Oncology Unit, Ospedale Cannizzaro, 94126 Catania, Italy; 5 Medical Oncology Unit, Ospedale Umberto I, 94019 Enna, Italy; 6 UOC Medical Oncology, AOUP Paolo Giaccone Palermo, 90127 Palermo, Italy; chiaramesi95@gmail.com (C.M.); doc.martinagreco@gmail.com (M.G.); dagatieleonora@gmail.com (E.D.); mariarosaria.valerio@unipa.it (M.R.V.); 7 Medical Oncology Unit, CDC Torina, 90100 Palermo, Italy; 8 Polistudium SRL, 20121 Milan, Italy; luca.giacomelli@polistudium.it; 9 Department of Precision Medicine in Critial Area (MEDPREC), University of Palermo, 90127 Palermo, Italy

**Keywords:** *BRCA* mutation, breast-conserving surgery, hereditary breast cancer, radiotherapy, systemic therapy, quality of life, psychosocial outcomes

## Abstract

Breast cancer associated with *BRCA1* and *BRCA2* mutations presents unique therapeutic challenges, traditionally favoring mastectomy due to concerns over recurrence and new primaries. However, evolving evidence and advances in multimodal therapy have reshaped this paradigm, positioning breast-conserving surgery (BCS) as a viable option for selected carriers. This narrative review synthesizes current data from meta-analyses, retrospective cohorts, and pivotal studies, including a multicenter analysis which affirmed oncologic equivalence between BCS and mastectomy when combined with radiotherapy and systemic therapy. While meta-analyses confirm higher local events following BCS, survival remains comparable, indicating that recurrence reflects genetic predisposition rather than surgical inadequacy. Optimized systemic treatments, including chemotherapy, endocrine therapy, risk-reducing salpingo-oophorectomy, and PARP inhibitors, further mitigate recurrence risk. Meanwhile, patient-centered outcomes favor BCS: studies consistently link it to improved body image, psychosocial well-being, and quality of life, especially for younger *BRCA* carriers. Fertility-preserving options remain viable, with evidence supporting the safety of pregnancy, breastfeeding, and assisted reproductive technologies in *BRCA*-mutated survivors. These findings support individualized surgical planning for *BRCA* carriers within multidisciplinary care, balancing oncologic safety, systemic strategies, and psychosocial priorities. BCS should be considered a standard option for well-selected patients in hereditary breast cancer management.

## 1. Introduction

Breast cancer is the most common malignancy among women worldwide, with 5–10% of cases attributable to hereditary predisposition, primarily due to pathogenic variants in the *BRCA1* and *BRCA2* genes [1,2]. These genes are crucial for homologous recombination repair, and their dysfunction results in genomic instability and substantially elevated lifetime risks of breast cancer, contralateral breast cancer, and ovarian cancer [3]. *BRCA*-associated breast cancers often exhibit distinct biological characteristics, including higher rates of triple-negative phenotype and early onset, necessitating tailored therapeutic approaches [4].

Historically, the detection of a *BRCA* mutation often prompted bilateral mastectomy as a therapeutic or prophylactic strategy, driven by concerns over local recurrence and new primaries in the conserved breast [3,5]. While risk-reducing mastectomy effectively lowers incidence, its significant physical and psychosocial impact has led to growing interest in breast-conserving surgery (BCS) for selected patients [3]. Current guidelines from the American Society of Clinical Oncology, the American Society for Radiation Oncology, and the Society of Surgical Oncology endorse BCS as an acceptable option for *BRCA* carriers meeting conventional criteria, if radiotherapy (RT) and systemic therapies are appropriately integrated [6].

Emerging evidence supports the oncologic feasibility of BCS in *BRCA* mutation carriers, with studies demonstrating comparable survival outcomes with mastectomy when adjuvant treatments are optimized [7,8]. Nevertheless, concerns persist regarding higher ipsilateral and contralateral breast cancer risks and the potential impact of RT in this genetically predisposed population [4,9]. Despite these increased risks, recent large cohort studies highlight that most *BRCA* carriers treated with BCS remain event-free over long-term follow-up and avoid subsequent mastectomy [9].

However, the suitability and safety of BCS in patients with *BRCA1* or *BRCA2* pathogenic variants remain relatively uncertain, reflecting heterogeneity in study designs, follow-up durations, and evolving treatment paradigms [10]. Furthermore, the expanding availability of genetic testing and the introduction of targeted therapies, such as poly(ADP-ribose) polymerase (PARP) inhibitors, underscore the need to re-examine surgical decision-making within a modern multidisciplinary context, considering fertility preservation [6,9,11].

Given these factors, there is a pressing need to synthesize current evidence on BCS for *BRCA* mutation carriers, critically appraise its oncologic and quality-of-life (QoL) implications, and clarify areas where knowledge gaps persist. This review aims to provide a comprehensive evaluation of the literature to inform clinical decision-making and guide personalized management strategies for this high-risk population.

## 2. Search Strategy

For this narrative review, relevant studies were identified through targeted searches of the PubMed, Scopus, and Web of Science databases. A Mac Microsoft Word, Version 16.102.2 (25102623) licenced to V.G. was employed to write the paper. We used a combination of free-text keywords and Medical Subject Headings (MeSH) to capture a comprehensive set of relevant articles. The core search terms included “*BRCA* mutations”, “breast-conserving surgery”, “breast-conserving therapy”, “mastectomy”, “local recurrence”, and “survival outcomes”. Boolean operators (AND, OR) were used to refine the search strategy. We included articles published up to August 2025. Only English-language publications were considered. This exclusion was based on concerns about potential translation inaccuracies and interpretation variability, which may introduce bias in a narrative synthesis. References cited in the retrieved articles were screened manually to identify additional relevant sources.

## 3. Evidence from Meta-Analyses

Systematic reviews and meta-analyses provide the highest-level evidence currently available on the comparative safety of BCS versus mastectomy in *BRCA* mutation carriers. Given the absence of randomized controlled trials in this setting, these pooled analyses are critical for informing surgical decision-making, offering insights into long-term local recurrence risk, contralateral breast cancer incidence, and survival outcomes (Table 1) [7,8,12,13].

### 3.1. Local Recurrence Risk

Several meta-analyses have consistently reported that BCS in *BRCA* mutation carriers is associated with an increased risk of local recurrence compared with mastectomy. Nara et al. synthesized data from 13 studies including 701 *BRCA* carriers and 4788 controls, demonstrating a significantly higher rate of ipsilateral breast tumor recurrence (IBTR) in carriers undergoing BCS (risk ratio [RR] 1.59; 95% CI, 1.25–2.02; *p* < 0.001), with the effect more pronounced at extended follow-up (≥7 years: RR 1.50; ≥10 years: RR 1.60) [12]. Davey et al., in the largest meta-analysis to date (*n* = 3807 carriers across 23 studies), reported a pooled hazard ratio (HR) of 4.54 (95% CI, 2.77–7.42) for locoregional recurrence (LRR) after BCS compared with mastectomy, with cumulative LRR rates of 14.7%, 15.5%, and 27.5% at 5, 10 and 15 years for BCS versus 4.8%, 4.7%, and 6.2% for mastectomy [13]. Similarly, Co et al. documented pooled ipsilateral recurrence rates of 8.2% at 5 years, 15.5% at 10 years, and 23% at 15 years for BCS, compared with 3.4%, 4.9% and 6.4% for mastectomy [7].

### 3.2. Contralateral Breast Cancer and Survival Outcomes

Despite increased local recurrence risk, survival outcomes appear unaffected. Wang et al. pooled four studies comprising 1254 *BRCA*-mutated patients [8]. They confirmed no significant differences between BCS and mastectomy in disease-free survival (DFS), metastasis-free survival, breast cancer-specific survival, or overall survival (OS), with HRs consistently near unity (e.g., OS: HR 1.02; 95% CI, 0.77–1.36) (Wang 2022 [8]). Davey et al. similarly reported equivalent 15-year OS between BCS (22.2% mortality) and mastectomy (23.9% mortality; HR 1.10; 95% CI, 0.72–1.69) [13]. Importantly, Nara et al. performed only a qualitative synthesis of OS data across three cohort studies, reporting no evidence of deterioration in survival among *BRCA* carriers treated with BCS [12]. Co et al. corroborated these findings, demonstrating overlapping 15-year OS rates (83.6% for BCS vs. 83.2% for mastectomy) and equivalent contralateral breast cancer risk (HR 1.51; 95% CI, 0.44–5.11) [7].

All meta-analyses emphasize the absence of randomized trials directly comparing BCS and mastectomy in *BRCA* carriers, reflecting ethical and logistical barriers to such studies. Consequently, available evidence derives from retrospective designs subject to selection bias, variability in systemic therapy use, and evolving surgical practices [7,13]. Nonetheless, the consistent observation of survival equivalence across pooled studies supports BCS as an oncologically safe alternative for selected *BRCA* carriers, if patients receive careful counseling on recurrence risk and undergo long-term surveillance.

Taken together, these meta-analyses establish the foundation for current understanding of BCS outcomes in *BRCA* carriers and highlight the need to examine complementary evidence from large retrospective cohort studies, which provide granular insights into real-world practice patterns, treatment era effects, and long-term follow-up.

## 4. Retrospective and Historical Cohort Studies in *BRCA* Mutation Carriers

In addition to meta-analyses, retrospective and historical cohort studies provide essential complementary evidence on the oncologic safety of BCS in *BRCA* mutation carriers. Spanning over four decades, these studies capture the evolution of clinical practice from the pre-genetic testing era to the modern framework of precision oncology. They collectively illustrate how improvements in early detection, systemic therapy, and genetic counseling have shaped patterns of recurrence and survival, offering a nuanced understanding of surgical decision-making for this high-risk group (Table 2) [9,10,14,15,16,17,18,19].

### 4.1. Historical Long-Term Data: Survival Equivalence Despite Higher In-Breast Events

Early studies established that although BCS was associated with higher IBTR, it did not compromise survival. In a multi-institutional cohort of 655 carriers with stage I–III breast cancer, Pierce et al. reported a 15-year IBTR risk of 23.5% following BCS compared with 5.5% after mastectomy (*p* < 0.0001), while 10-year OS was virtually identical (92.1% for BCS vs. 91.8% for mastectomy) [14]. Detailed pathological reviews showed that most IBTRs were new primary tumors rather than true recurrences, reflecting the inherent predisposition of *BRCA* carriers to develop new malignancies rather than a failure of local therapy.

Nilsson et al. similarly analyzed 162 Swedish carriers over a mean follow-up of nearly 13 years and reported a 15-year cumulative IBTR rate of 32% for BCS compared with 9% for mastectomy (HR 2.9; 95% CI, 1.1–7.8), with OS equivalence (HR 1.2; 95% CI, 0.5–2.9) [15]. These early findings, arising before widespread use of adjuvant chemotherapy or endocrine therapy in carriers, set the precedent that survival equivalence can be maintained despite increased local events.

### 4.2. Transitional Evidence: The Mitigating Impact of Systemic Therapy and Prophylactic Strategies

Later cohorts demonstrated that adjuvant systemic therapies and prophylactic interventions narrowed the recurrence gap between surgical modalities. Van den Broek et al. assessed 191 *BRCA1* carriers diagnosed between 1970 and 2003 and found comparable 10-year IBTR rates for BCS (7.3%) and non-carriers (7.9%), with no OS difference (HR 0.80; 95% CI, 0.42–1.51) [16]. Gentile et al. reinforced these findings in a series of 124 carriers treated between 2008 and 2018, reporting no significant differences in DFS or OS between BCS and mastectomy, while noting improved distant DFS and OS among women who underwent bilateral salpingo-oophorectomy (BSO) [17]. These results underscore how risk-reducing surgery and optimized adjuvant treatments have reshaped outcomes in carriers over the past two decades.

### 4.3. Contemporary Cohorts: Outcomes in the Era of Precision Oncology

Recent studies, conducted within the context of routine germline testing, intensified surveillance, and modern systemic therapy, have provided the strongest reassurance of BCS safety in *BRCA* carriers. Shubeck et al. analyzed 395 carriers treated between 2006 and 2015, finding no differences in 10-year LRR (7.3% after BCS vs. 6.3% after mastectomy) or OS (88.8% vs. 87.5%) [18]. Wanis et al., in a single-institution series of 172 BCS-treated carriers with a median follow-up of 11.8 years, reported 10-year ipsilateral and contralateral breast event rates of 12.2% and 21.3%, respectively. Most patients avoided subsequent bilateral mastectomy, and the OS rate was 88.5% [9]. Mahiou et al. corroborated these findings in a French registry study (*n* = 104), observing no significant differences in OS (HR 1.49; 95% CI, 0.76–2.93) or recurrence-free survival (RFS; HR 1.40; 95% CI, 0.81–2.40) between BCS and mastectomy [19].

### 4.4. The Lee et al. Multicenter Study: High-Quality Evidence in the Genomic Era

Among contemporary analyses, the multicenter study by Lee et al. represents the most comprehensive evaluation of surgical outcomes in *BRCA* carriers to date [10]. This study enrolled 575 carriers diagnosed between 2008 and 2015 across multiple high-volume centers. It used rigorous propensity score matching to control for confounding factors, including age, tumor size, nodal status, hormone receptor subtype, and systemic therapy. Following matching, 159 patients per group were compared, with a median follow-up of 8.3 years.

Lee et al. reported no significant differences in locoregional RFS (HR 0.96; 95% CI, 0.36–2.59), distant RFS (HR 0.62; 95% CI, 0.28–1.38), RFS (HR 0.63; 95% CI, 0.33–1.22), or OS (HR 0.82; 95% CI, 0.34–1.98) between BCS and mastectomy [10]. Importantly, subgroup analyses stratified by *BRCA* mutation type (*BRCA1* vs. *BRCA2*) and tumor biology (triple-negative vs. hormone receptor-positive) confirmed these findings, supporting the applicability of BCS across biologically distinct subgroups within the *BRCA*-mutated population. Notably, while 14.2% of patients developed contralateral breast cancer during follow-up, this was independent of initial surgical approach and consistent with known contralateral risk profiles in this population.

A key strength of this study lies in the integration of systemic therapies: over 85% of patients received chemotherapy, and 80% of eligible patients received endocrine therapy, reflecting modern treatment standards. The inclusion of robust RT further mirrors current practice, addressing earlier concerns about radio resistance in *BRCA* carriers. Together, these factors underscore the relevance of this study in informing contemporary decision-making.

These data collectively illustrate an evolving trajectory: while early studies confirmed survival equivalence despite higher in-breast events, more recent cohorts show that with modern systemic therapies, optimized RT, and rigorous surveillance, recurrence risks associated with BCS are reduced and survival remains unaffected. The study by Lee et al. offers compelling evidence that, in the genomic era, BCS is an oncologically safe alternative to mastectomy for appropriately selected *BRCA* carriers, provided that vigilant follow-up and complementary risk-reducing strategies are implemented [10]. Persistent contralateral breast cancer risk remains a defining feature of this population, underscoring the importance of ongoing surveillance and shared decision-making when selecting local treatment.

## 5. Impact of RT and Locoregional Treatment Modalities

RT remains a cornerstone of breast-conserving therapy and plays a crucial role in optimizing local control for *BRCA* mutation carriers. Historically, concerns regarding increased radiosensitivity in *BRCA*-associated breast cancers raised questions about the safety and efficacy of RT. However, RT has been shown to be safe and effective in this population, with no evidence of increased toxicity or adverse survival impact.

More recent data have reinforced and refined this understanding, particularly in the context of evolving surgical techniques such as skin-sparing and nipple-sparing mastectomy (SSM/NSM). Bernstein-Molho et al. analyzed 484 breast tumors in 464 *BRCA* carriers treated between 2006 and 2022. Post-mastectomy RT significantly reduced LRR rates compared with mastectomy alone (1.25% vs. 13%, *p* = 0.003), despite the post-mastectomy RT group presenting with more advanced disease [20]. By contrast, mastectomy without RT, often performed in early-stage patients or those identified via genetic testing after lumpectomy, was associated with elevated early LRR rates, particularly within the first 2 years post-surgery. These results support the role of RT in eradicating residual microscopic disease in high-risk carriers, even following mastectomy with reconstructive techniques that may leave behind residual breast tissue.

Further insights come from Moshe et al., who investigated 255 patients (127 *BRCA* and 128 non-*BRCA*) undergoing SSM/NSM with or without RT [21]. They reported that patients not receiving RT had significantly higher LRR rates (15.3%) compared with those treated with RT (0%), independent of *BRCA* status. Multivariable analysis confirmed that RT was an independent protective factor against LRR (HR 0.67; 95% CI, 0.31–0.91). Interestingly, the study highlighted the early timing of recurrences in non-irradiated patients, suggesting residual breast tissue within skin flaps or dermal lymphatics as a plausible source of failure. These data underscore the critical importance of meticulous surgical planning and the integration of RT in cases where residual microscopic disease is likely.

Collectively, these findings emphasize that RT is integral to modern locoregional management in *BRCA* carriers. In the genomic era, its use extends beyond traditional indications to address the interplay between advanced surgical approaches (e.g., SSM/NSM), reconstruction techniques, and intrinsic genetic risk. Importantly, RT provides durable local control without increasing contralateral breast cancer risk or compromising survival, reinforcing its role within a multimodal treatment paradigm tailored to hereditary breast cancer biology.

## 6. Integration with Systemic and Risk-Reducing Strategies

Adjuvant systemic therapies and prophylactic interventions are key determinants of oncologic outcomes for *BRCA* mutation carriers undergoing BCS, complementing local therapy in reducing recurrence risk and improving survival. Modern management paradigms integrate chemotherapy, endocrine therapy, and risk-reducing BSO, alongside the emerging role of PARP inhibitors, to address the intrinsic biology of *BRCA*-associated breast cancer.

The prognostic value of risk-reducing BSO has been consistently demonstrated. Gentile et al. retrospectively analyzed 124 *BRCA* mutation carriers treated between 2008 and 2018, finding no difference in DFS or OS between BCS and mastectomy, but significantly improved distant DFS (*p* = 0.033) and OS (*p* = 0.040) among women who underwent BSO [17]. These results underscore the dual impact of systemic endocrine modulation and ovarian ablation in reducing recurrence risk, particularly in hormone receptor-positive carriers.

Chemotherapy and endocrine therapy remain central to modern BCS outcomes. In the contemporary series by Shubeck et al., involving 395 *BRCA* carriers treated between 2006 and 2015, receipt of adjuvant systemic therapy was high (74% chemotherapy, 54% endocrine therapy), and no differences were observed in LRR (7.3% for BCS vs. 6.3% for mastectomy) or OS (10-year OS: 88.8% vs. 87.5%) [18]. These findings suggest that optimized systemic therapy mitigates local recurrence risk and supports oncologic equivalence between surgical approaches in *BRCA* carriers.

The single-institution cohort by Wanis et al. extended these findings to long-term follow-up. Among 172 carriers treated with BCS over four decades, the 10-year OS was 88.5% (95% CI, 83.1–94.2%), with ipsilateral and contralateral breast cancer event risks of 12.2% and 21.3%, respectively [9]. Importantly, adjuvant RT (HR 0.09; 95% CI, 0.02–0.54) and BSO (HR 0.29; 95% CI, 0.09–0.90) were independently associated with lower ipsilateral recurrence risk. Endocrine therapy showed a trend toward reduced contralateral breast events (HR 0.61; 95% CI, 0.31–1.20), while chemotherapy was associated with lower distant recurrence risk (HR 0.48; 95% CI, 0.22–0.96). These results reinforce the synergistic interplay between systemic and local interventions in hereditary breast cancer management.

Emerging targeted therapies further reshape this landscape. PARP inhibitors, particularly olaparib, have demonstrated substantial benefits in *BRCA*-associated early breast cancer, with the OlympiA trial showing significant improvements in invasive DFS and distant RFS in high-risk carriers receiving adjuvant olaparib [9]. Although direct data on PARP inhibitors in the context of BCS are limited, their integration into standard adjuvant regimens is expected to further reduce recurrence risk, particularly in patients with residual disease or triple-negative tumors.

Collectively, these findings illustrate that the favorable outcomes of BCS in *BRCA* carriers are closely tied to modern systemic therapy utilization and prophylactic strategies (Table 3) [9,17,18]. The combination of optimized adjuvant treatment, BSO, and, increasingly, PARP inhibition provides a comprehensive multimodal framework that supports BCS as an oncologically sound option for appropriately selected carriers.

## 7. Pregnancy, Breastfeeding, and Assisted Reproductive Technologies in *BRCA*-Mutated Breast Cancer Survivors

Reproductive considerations represent a central component in the holistic management of young women with *BRCA*-mutated breast cancer. In recent years, a series of large-scale, collaborative studies have reshaped the evidence base surrounding the safety and feasibility of pregnancy, breastfeeding, and assisted reproductive technologies (ART) in this population. Collectively, these data reinforce the concept of personalized survivorship care and support the integration of fertility-preserving strategies and family planning in *BRCA*-mutated breast cancer management.

A recent large international cohort study assessed the safety of breastfeeding among 178 *BRCA1*/*2* carriers who delivered a child after breast cancer and for whom lactation data were available. In this cohort, 61.8% breastfed their child, with a median duration of 5 months. After a median follow-up of 7 years post-delivery, no significant differences were observed in DFS (adjusted HR 0.83; 95% CI, 0.49–1.41) or OS (adjusted HR 1.32; 95% CI, 0.31–5.66) between women who breastfed and those who did not. Furthermore, the cumulative incidence of locoregional or contralateral recurrences was numerically lower in the breastfeeding group (29% vs. 37%), though not statistically significant [22].

Assisted reproduction represents another critical concern, particularly for *BRCA* mutation carriers who may experience impaired ovarian reserve due to both genetic predisposition and oncologic treatment. A multicenter study by Magaton et al. examined outcomes in 543 *BRCA1*/*2* carriers who became pregnant after breast cancer, of whom 107 conceived through ART. Among these, 42.1% used oocytes or embryos cryopreserved at diagnosis, 30.8% underwent in vitro fertilization or ovulation induction after treatment, and 19.6% used donor oocytes. At a median follow-up of 5.2 years, the use of ART was not associated with worse DFS (adjusted HR 0.72; 95% CI, 0.39–1.34), providing reassuring evidence for the oncologic safety of ART in this setting [23].

Pregnancy-related outcomes were also comparable between the ART and non-ART groups. The live birth rate was 83.0% among ART users and 79.8% among those who conceived spontaneously. Most pregnancies in both groups were delivered at term (85.5% vs. 91.5%, respectively), and complication rates were low. Notably, a slightly higher incidence of miscarriage was observed in the ART group (11.3% vs. 8.8%), which may reflect older maternal age at conception in this subgroup [23].

In parallel, Perachino et al. explored reproductive outcomes following a diagnosis of pregnancy-associated breast cancer (PrBC) in *BRCA* carriers—a scenario historically associated with limited data. Among 282 patients diagnosed with PrBC, 68 experienced a subsequent pregnancy, with a 10-year cumulative incidence of 36.6%. The majority of pregnancies were full-term (75.9%) and without complications (74.1%). Importantly, no differences in DFS (adjusted HR 1.06; 95% CI, 0.49–2.31) or OS (adjusted HR 0.63; 95% CI, 0.19–2.05) were noted between women with or without subsequent pregnancy, suggesting that a history of PrBC does not preclude safe future childbearing in this high-risk group [24].

Finally, these findings build upon a growing body of work, including earlier observational data by Blondeaux et al., which have consistently shown that *BRCA* mutation status should not, per se, limit reproductive planning. For example, factors such as nulliparity at diagnosis, hormone receptor status, and time to conception were explored in these cohorts and support a nuanced approach to counseling [25].

Altogether, these studies converge to reinforce that pregnancy, breastfeeding, and ART can be safely considered in selected *BRCA*-mutated breast cancer survivors. These findings lend further support to the paradigm shift toward personalized treatment strategies and away from blanket recommendations for risk-reducing mastectomy or reproductive deferral. In this context, breast-conserving strategies gain additional relevance—not only for their oncologic equivalence but also for preserving body image and function in women who may wish to breastfeed or consider future pregnancy. Importantly, these data provide clinicians with much-needed reassurance to support shared decision-making in a setting where reproductive autonomy and oncologic safety must be balanced.

## 8. Psychosocial and Patient-Centered Outcomes

Specific evidence addressing psychosocial and QoL outcomes in *BRCA* mutation carriers undergoing BCS remains limited. Consequently, insights must largely be extrapolated from recent prospective and cross-sectional studies conducted in broader breast cancer populations, where breast-conserving approaches consistently demonstrate favorable patient-reported outcomes compared with mastectomy and reconstruction. These findings are particularly relevant given the oncologic equivalence of BCS and mastectomy in *BRCA* carriers and the critical importance of patient-centered decision-making in this context.

Recent studies have underscored the QoL advantages of BCS over more extensive surgical approaches. In a large propensity score–matched study of over 2600 patients, Kim et al. reported that women undergoing BCS scored significantly higher on BREAST-Q domains—including satisfaction with breasts, psychosocial and sexual well-being—throughout 3 years of follow-up compared with those undergoing postmastectomy reconstruction (*p* < 0.05 for all) [26]. Similarly, Liu et al., in a prospective cohort of 471 patients, observed that BCS was associated with the highest absolute postoperative scores across multiple health-related QoL dimensions, including breast satisfaction and psychosocial and physical well-being (*p* < 0.01), while mastectomy patients exhibited greater declines in pain and functional domains [27]. Importantly, younger age, which is common among *BRCA* mutation carriers, was independently associated with higher levels of postoperative anxiety, depression, and pain, suggesting that hereditary breast cancer patients may face amplified psychosocial challenges regardless of surgical choice [27].

Complementary cross-sectional data reinforce these trends. Tomita et al. reported that patients treated with BCS achieved significantly better scores for breast satisfaction (mean 58.3) and sexual well-being (mean 45.8) than those undergoing mastectomy (41.8 and 29.9, respectively). However, psychosocial well-being was highest in reconstruction cohorts, reflecting the complex interplay between body image, surgical choice, and psychological adaptation [28]. Furthermore, van Zeelst et al. demonstrated that integration of oncoplastic refinements, such as perforator flap reconstruction, can enhance esthetic outcomes within BCS, with median BREAST-Q scores of 87/100 for psychosocial well-being and 82/100 for breast satisfaction at 17 months, alongside low complication rates and high outpatient feasibility [29].

Although not *BRCA*-specific, these recent studies suggest that BCS may confer measurable QoL advantages relative to mastectomy, particularly in terms of breast satisfaction, psychosocial adjustment, and functional well-being. For *BRCA* carriers, these benefits must be weighed against their distinct cancer risk profiles and the need for long-term surveillance. Nonetheless, they underscore the importance of incorporating patient-reported outcomes into shared decision-making. Given that younger age and intensive adjuvant therapy are linked to higher distress [27], structured psychosocial support and counseling may be particularly crucial for hereditary breast cancer patients selecting BCS.

## 9. BCS in *BRCA* Mutation Carriers: A Paradigm Shift Toward Individualized Care

The surgical management of breast cancer in *BRCA* mutation carriers is undergoing a transformative shift. Historically, mastectomy was considered the default approach due to concerns about elevated recurrence risks and the propensity for new primary tumors in this high-risk group. However, recent evidence demonstrates that with the integration of modern RT, systemic therapy, and vigilant surveillance, BCS can be considered an oncologically safe option for appropriately selected carriers [1,10,13].

This evolution reflects broader advances in multidisciplinary care. Improved systemic treatments, including chemotherapy, endocrine therapy, and more recently PARP inhibitors, have significantly contributed to reducing recurrence risk and improving long-term outcomes in *BRCA* carriers [1,9]. Furthermore, risk-reducing strategies such as BSO remain integral in comprehensive management, particularly in hormone receptor-positive disease [17]. These developments collectively redefine the balance between local and systemic control in hereditary breast cancer.

Beyond oncologic safety, psychosocial and QoL outcomes are becoming central to surgical decision-making. Studies in broader breast cancer populations consistently show that BCS is associated with greater satisfaction with breast appearance, improved body image, and more favorable psychosocial well-being compared with mastectomy and reconstruction [2]. Although such data in *BRCA* carriers are limited, these insights are relevant and likely applicable, given similar surgical contexts. For many women, retaining their breasts may mitigate the psychological burden associated with hereditary cancer, reducing feelings of loss and improving sexual and emotional adjustment. These potential benefits highlight the importance of integrating psychosocial considerations into surgical counseling and tailoring choices not solely to risk reduction, but also to patient-reported priorities.

Emerging data also affirm the safety of pregnancy and breastfeeding in *BRCA* carriers after treatment. Recent international studies show no increased risk of recurrence or mortality following either outcome, and breastfeeding was not associated with adverse oncologic events [22,24]. These findings are especially relevant for women who pursue BCS, which may allow for future lactation.

Similarly, ART appears safe in this population. A multicenter study reported no significant differences in DFS between women conceiving through ART and those with spontaneous pregnancies [23], further supporting reproductive autonomy in survivorship care.

Together, these findings endorse a more personalized surgical approach that considers not only oncologic risk but also reproductive goals and long-term QoL. For *BRCA* carriers, particularly younger patients, BCS can offer oncologic equivalence with potential functional and psychosocial advantages.

Nonetheless, challenges remain. The persistent risk of contralateral breast cancer necessitates lifelong enhanced surveillance and underscores the importance of comprehensive genetic counseling and psychosocial support [9]. Furthermore, differences in tumor biology and systemic therapy response between *BRCA1* and *BRCA2* carriers require individualized strategies [10].

Importantly, not all *BRCA* mutation carriers face the same risk profile or respond to treatment similarly. Breast cancer patients carrying a *BRCA* mutation who may safely avoid mastectomy can consider breast-conserving surgery followed by radiation therapy. This option may be particularly suitable for *BRCA2* carriers, as several studies suggest that BCS with radiation may yield comparable or even favorable outcomes relative to mastectomy. In contrast, *BRCA1* carriers are known to have a higher risk of contralateral breast cancer, which may increase the likelihood of recommending prophylactic mastectomy.

Other key selection criteria include tumor characteristics—such as size (T stage), nodal involvement (N status), grade, and hormone receptor profile. Luminal tumors, typically associated with hormone receptor positivity, often have better prognoses and may be more amenable to breast conservation, while triple-negative cancers, more common in *BRCA1* carriers, may favor a more aggressive surgical approach. Furthermore, individual psychological tolerance for risk plays a critical role. Some patients may prioritize maximum risk reduction and prefer mastectomy, while others may find the trade-off between oncologic safety and body preservation acceptable in choosing BCS. Looking forward, BCS for *BRCA* carriers represents a paradigm shift from uniform recommendations toward personalized, patient-centered care. While a growing body of retrospective evidence suggests that breast conservation may be considered as part of multimodal treatment planning for appropriately selected individuals, this must be balanced against emerging data indicating a potential survival advantage with risk-reducing mastectomy. Notably, Blondeaux et al. (2025) [25] reported improved DRFS and OS with contralateral risk-reducing mastectomy in young *BRCA* carriers, highlighting the importance of individualized decision-making based on both oncologic and patient-centered factors.

Ultimately, surgical choice in *BRCA* mutation carriers must balance oncologic safety, systemic therapy integration, and the profound psychological implications of breast loss or preservation. By embedding BCS within comprehensive multidisciplinary care, including genetic counseling, risk-reduction planning, and psychosocial support, we can better align treatment with both medical evidence and individual patient values. In this context, incorporating formal psychological assessment and structured support into preoperative counseling is essential to help patients navigate the complex emotional and decisional aspects of choosing between BCS and mastectomy.

## 10. Conclusions

BCS is increasingly recognized as a valid and oncologically acceptable option for selected *BRCA* mutation carriers, particularly when delivered within a comprehensive, multidisciplinary framework. Accumulating evidence, though largely retrospective, consistently supports equivalent long-term survival outcomes between BCS and mastectomy, especially when radiotherapy and systemic therapies are appropriately integrated.

Importantly, BCS may offer meaningful advantages in terms of body image, quality of life, and reproductive planning—factors that are particularly relevant for younger patients navigating the complex implications of hereditary breast cancer. However, we recognize that most existing QoL data are derived from studies involving non-*BRCA* populations. While these findings provide useful context, they should be interpreted with caution when applied to *BRCA* carriers, given their distinct psychosocial and clinical challenges. This underscores the need for future *BRCA*-specific research to inform patient-centered care more accurately. At the same time, we acknowledge the limitations inherent in the current bulk of evidence, including the retrospective nature of most studies and heterogeneity in design and follow-up. The recent international cohort study by Blondeaux et al. (2025) [25], which demonstrated improved distant recurrence-free and overall survival with risk-reducing mastectomy in young *BRCA* carriers, provides important prospective data that must be considered in surgical counseling.

Taken together, these findings reinforce the need for nuanced, patient-centered decision-making that balances oncologic safety with psychosocial well-being and personal values. As evidence continues to evolve, BCS should be presented as a standard treatment option—not a compromise—for appropriately selected *BRCA* mutation carriers.

## Figures and Tables

**Table 1:** Summary of key meta-analyses comparing BCS and mastectomy in *BRCA* mutation carriers.

Study (Year)	Design	*n* (*BRCA* Carriers)	Follow-Up (Median)	LRR/IBTR Risk (BCS vs. Mastectomy)	OS Difference	Contralateral BC Risk
Nara 2022 [12]	Meta-analysis	701	≥10 years	RR 1.59 (95% CI, 1.25–2.02)	Qualitative: No deterioration in OS	Not assessed
Davey 2021 [13]	Meta-analysis	3807	Up to 15 years	HR 4.54 (95% CI, 2.77–7.42)	HR 1.10 (95% CI, 0.72–1.69)	HR 1.51 (95% CI, 0.44–5.11)
Co 2020 [7]	Systematic review	2157	5–15 years	LRR 8.2%, 15.5%, 23% vs. 3.4%, 4.9%, 6.4%	5-, 10-, 15-year OS comparable	Not reported
Wang 2022 [8]	Meta-analysis	1254	Variable	HR 3.84 (95% CI, 2.38–6.20)	HR 1.02 (95% CI, 0.77–1.36)	Not significant

BC, breast cancer; BCS, breast-conserving surgery; HR, hazard ratio; IBTR, ipsilateral breast tumor recurrence; LRR, locoregional recurrence; OS, overall survival; RR, risk ratio.

**Table 2:** Summary of retrospective and historical cohort studies evaluating BCS versus mastectomy in *BRCA* mutation carriers.

Study (Year)	Design	*n* (*BRCA* Carriers)	Follow-Up (Median)	Key Findings
Pierce 2010 [14]	Multi-institutional cohort	655 (302 BCS; 353 mastectomy)	8–15 years	15-year IBTR: 23.5% (BCS) vs. 5.5% (mastectomy); no OS difference (10-year OS: 92.1% vs. 91.8%); most IBTRs were new primaries.
Nilsson 2014 [15]	Single-institution cohort	162 (45 BCS; 117 mastectomy)	12.9 years	15-year IBTR: 32% (BCS) vs. 9% (mastectomy); HR 2.9 (95% CI, 1.1–7.8); OS equivalent between groups.
van den Broek 2019 [16]	Population-based cohort	191 *BRCA1* carriers (mixed control cohort)	10 years	IBTR after BCS: 7.3% (*BRCA1*) vs. 7.9% (non-carriers); OS: HR 0.80 (95% CI, 0.42–1.51); comparable outcomes with non-carriers.
Gentile 2022 [17]	Single-institution cohort	124 (69 BCS; 55 mastectomy)	~10 years	No difference in DFS, distant DFS, or OS; BSO significantly improved distant DFS and OS (*p* = 0.033 and *p* = 0.040).
Shubeck 2022 [18]	Single-institution cohort	395 (149 BCS; 246 mastectomy)	7.9 years	10-year LRR: 7.3% (BCS) vs. 6.3% (mastectomy); OS: 88.8% vs. 87.5%; contralateral BC risk higher after unilateral surgery.
Wanis 2024 [9]	Single-institution cohort	172 (all BCS)	11.8 years	10-year IBTR: 12.2%; contralateral BC: 21.3%; OS: 88.5%; majority remained bilateral mastectomy-free.
Mahiou 2024 [19]	Registry-based cohort	104 (69 BCS; 35 mastectomy)	~10 years	No OS difference (HR 1.49; *p* = 0.25); no difference in RFS (HR 1.40; *p* = 0.22).
Lee 2025 [10]	Multicenter propensity-matched cohort	575 (159 matched pairs)	8.3 years	No difference in locoregional RFS (HR 0.96), distant RFS (HR 0.62), RFS (HR 0.63), or OS (HR 0.82); contralateral BC: 14.2%; outcomes consistent across *BRCA1/2* subgroups.

BC, breast cancer; BCS, breast-conserving surgery; BSO, bilateral salpingo-oophorectomy; DFS, disease-free survival; HR, hazard ratio; IBTR, ipsilateral breast tumor recurrence; LRR, locoregional recurrence; OS, overall survival; RFS, recurrence-free survival.

**Table 3:** Summary of studies evaluating systemic therapy and risk-reducing interventions in *BRCA* mutation carriers undergoing BCS.

Study (Year)	Design	N (*BRCA* Carriers)	Key Systemic/Risk- Reducing Interventions	Key Outcomes
Gentile 2022 [17]	Single-institution cohort	124 (69 BCS; 55 mastectomy)	BSO (58.1%); adjuvant chemotherapy and endocrine therapy per subtype	No DFS (*p* = 0.39) or OS (*p* = 0.265) difference between BCS and mastectomy; BSO improved distant DFS (*p* = 0.033) and OS (*p* = 0.040).
Shubeck 2022 [18]	Single-institution cohort	395 (149 BCS; 246 mastectomy)	Chemotherapy (74%), endocrine therapy (54%), RT (65% BCS)	10-year LRR: 7.3% (BCS) vs. 6.3% (mastectomy); 10-year OS: 88.8% vs. 87.5%; systemic therapy supported equivalence.
Wanis 2024 [9]	Single-institution cohort	172 (all BCS)	Adjuvant RT (100%), BSO (54%), endocrine therapy, chemotherapy; PARP inhibitor context (OlympiA)	10-year OS: 88.5% (95% CI, 83.1–94.2); ipsilateral recurrence: 12.2%; contralateral events: 21.3%; RT (HR 0.09; 95% CI, 0.02–0.54) and BSO (HR 0.29; 95% CI, 0.09–0.90) reduced ipsilateral recurrence; endocrine therapy (HR 0.61; 95% CI, 0.31–1.20) lowered contralateral risk; chemotherapy (HR 0.48; 95% CI, 0.22–0.96) reduced distant recurrence.

BCS, breast-conserving surgery; BSO, bilateral salpingo-oophorectomy; DFS, disease-free survival; HR, hazard ratio; LRR, locoregional recurrence; OS, overall survival; PARP, poly(ADP-ribose) polymerase; RT, radiotherapy.

## Data Availability

No new data were created or analyzed in this study.

## References

[1] Edaily S., Abdel-Razeq H. (2022). Management Strategies of Breast Cancer Patients with *BRCA1* and *BRCA2* Pathogenic Germline Variants. Onco Targets Ther..

[2] Magnoni F., Sacchini V., Veronesi P., Bianchi B., Bottazzoli E., Tagliaferri V., Mazzotta E., Castelnovo G., Deguidi G., Rossi E.M.C. (2022). Surgical Management of Inherited Breast Cancer: Role of Breast-Conserving Surgery. Cancers.

[3] Cipolla C., Scandurra G., Sambataro D., Mesi C., Greco M., D’Agati E., Gebbia V., Valerio M.R. (2025). Risk-reducing mastectomy in healthy women with *BRCA* mutation: A narrative review. Expert. Rev. Anticancer. Ther..

[4] Vallard A., Magné N., Guy J.B., Espenel S., Rancoule C., Diao P., Deutsch E., Rivera S., Chargari C. (2019). Is breast-conserving therapy adequate in BRCA 1/2 mutation carriers? The radiation oncologist’s point of view. Br. J. Radiol..

[5] Terribile D.A., Mason E.J., Murando F., DILeone A., Sanchez A.M., Scardina L., Magno S., Franco A., D’Archi S., Natale M. (2021). Surgical management of BRCA pathogenic variant carriers with breast cancer: A recent literature review and current state of the art. Minerva Surg..

[6] Tung N.M., Boughey J.C., Pierce L.J., Robson M.E., Bedrosian I., Dietz J.R., Dragun A., Gelpi J.B., Hofstatter E.W., Isaacs C.J. (2020). Management of Hereditary Breast Cancer: American Society of Clinical Oncology, American Society for Radiation Oncology, and Society of Surgical Oncology Guideline. J. Clin. Oncol..

[7] Co M., Liu T., Leung J., Li C.H., Tse T., Wong M., Kwong A. (2020). Breast Conserving Surgery for BRCA Mutation Carriers-A Systematic Review. Clin. Breast Cancer.

[8] Wang C., Lin Y., Zhu H., Zhou Y., Mao F., Huang X., Zhou X., Cao X., Sun Q. (2022). Breast-conserving therapy for breast cancer with BRCA mutations: A meta-analysis. Breast Cancer.

[9] Wanis K.N., Kuerer H.M., Sun S.X., Hunt K.K., Glencer A.C., Teshome M., Lucci A., Weiser R., Johnson H., Smith B.D. (2024). Clinical Outcomes for BRCA Pathogenic Variant Carriers With Breast Cancer Undergoing Breast Conservation. JAMA Netw. Open.

[10] Lee J., Ryu J.M., Kim H.K., Park H.S., Kang B., Ahn S.G., Chung M.S., Shin S.H., Go J., Kim S. (2025). Long-Term Oncologic Outcome of Breast-Conserving Treatment in Patients With Breast Cancer With BRCA Variants. JAMA Netw. Open.

[11] Magaton I.M., Arecco L., Mariamidze E., Jankovic K., Stana M., Buzzatti G., Trevisan L., Scavone G., Ottonello S., Fregatti P. (2024). Fertility and Pregnancy-Related Issues in Young *BRCA* Carriers With Breast Cancer. Breast Cancer.

[12] Nara M., Ishihara S., Kitano A., Tamura N., Aruga T., Kobayashi D., Nakamura S., Yamauchi H. (2022). Does breast-conserving surgery with radiotherapy in *BRCA*-mutation carriers significantly increase ipsilateral breast tumor recurrence? A systematic review and meta-analysis. Breast Cancer.

[13] Davey M.G., Davey C.M., Ryan É.J., Lowery A.J., Kerin M.J. (2021). Combined breast conservation therapy versus mastectomy for *BRCA* mutation carriers—A systematic review and meta-analysis. Breast.

[14] Pierce L.J., Phillips K.A., Griffith K.A., Buys S., Gaffney D.K., Moran M.S., Haffty B.G., Ben-David M., Kaufman B., Garber J.E. (2010). Local therapy in BRCA1 and BRCA2 mutation carriers with operable breast cancer: Comparison of breast conservation and mastectomy. Breast Cancer Res. Treat..

[15] Nilsson M.P., Hartman L., Kristoffersson U., Johannsson O.T., Borg A., Henriksson K., Lanke E., Olsson H., Loman N. (2014). High risk of in-breast tumor recurrence after *BRCA1*/2-associated breast cancer. Breast Cancer Res. Treat..

[16] van den Broek A.J., Schmidt M.K., van’t Veer L.J., Oldenburg H.S.A., Rutgers E.J., Russell N.S., Smit V.T.H.B.M., Voogd A.C., Koppert L.B., Siesling S. (2019). Prognostic Impact of Breast-Conserving Therapy Versus Mastectomy of *BRCA1*/2 Mutation Carriers Compared with Noncarriers in a Consecutive Series of Young Breast Cancer Patients. Ann. Surg..

[17] Gentile D., Losurdo A., Sagona A., Zuradelli M., Gatzemeier W., Barbieri E., Testori A., Errico V., Bianchi P., Biondi E. (2022). Surgical management of *BRCA*-mutation carriers: A single institution experience. Eur. J. Surg. Oncol..

[18] Shubeck S., Sevilimedu V., Berger E., Robson M., Heerdt A.S., Pilewskie M.L. (2022). Comparison of Outcomes Between *BRCA* Pathogenic Variant Carriers Undergoing Breast-Conserving Surgery Versus Mastectomy. Ann. Surg. Oncol..

[19] Mahiou K., Jankowski C., Vincent L., Costaz H., Padeano M.M., Mamguem A., Dabakuyo S., Coutant C. (2024). Impact of breast surgical procedure on survival in BRCA mutated patients with invasive breast cancer: Mastectomy versus conservative treatment. J. Gynecol. Obstet. Hum. Reprod..

[20] Bernstein-Molho R., Haisraely O., Galper S., Abu-Shhada N., Nili Gal-Yam E., Menes T.S., Poortmans P., Kaidar-Person O. (2025). The impact of locoregional treatment on ipsilateral breast cancer recurrence and outcomes in carriers of *BRCA1*/2 pathogenic variants. Breast Cancer Res. Treat..

[21] Moshe N., Haisraely O., Globus O., Faermann R., Abu-Shehada N., Anaby D., Gal Yam E., Balint Lahat N., Galper S., Menes T. (2025). Breast cancer outcomes after skin- and nipple-sparing mastectomy in BRCA pathogenic mutation carriers versus non-BRCA carriers. Radiother. Oncol..

[22] Blondeaux E., Delucchi V., Mariamidze E., Bernstein-Molho R., Frank S., Ferrari A., Linn S., Jeong Kim H., Agostinetto E., Paluch-Shimon S. (2025). Breastfeeding after breast cancer in young *BRCA* carriers. J. Natl. Cancer Inst..

[23] Magaton I.M., Blondeaux E., Hamy A.S., Linn S., Bernstein-Molho R., Peccatori F.A., Ferrari A., Carrasco E., Paluch-Shimon S., Agostinetto E. (2025). Assisted reproductive technology in young BRCA carriers with a pregnancy after breast cancer: An international cohort study. Eur. J. Cancer.

[24] Perachino M., Blondeaux E., Delucchi V., Bernstein Molho R., Frank S., Paluch-Shimon S., Linn S., Di Meglio A., Peccatori F., Moore H.C.F. (2025). Safety of having a subsequent pregnancy after prior diagnosis of breast cancer during pregnancy in young BRCA carriers. ESMO Open.

[25] Blondeaux E., Sonnenblick A., Agostinetto E., Bas R., Kim H.J., Franzoi M.A., Bernstein-Molho R., Linn S., Kwong A., Pogoda K. (2025). Association between risk-reducing surgeries and survival in young BRCA carriers with breast cancer: An international cohort study. Lancet Oncol..

[26] Kim M., Tadros A.B., Boe L.A., Vingan P., Allen R.J., Mehrara B.J., Morrow M., Nelson J.A. (2024). Breast-Conserving Therapy Versus Postmastectomy Breast Reconstruction: Propensity Score-Matched Analysis. Ann. Surg. Oncol..

[27] Liu C., Beresford A., Saleeb M., Liu G., Crump T., Warburton R., Pao J.S., Dingee C.K., Bazzarelli A., Sutherland J.M. (2025). Preoperative and Postoperative Change in Patient-Reported Health-Related Quality of Life Outcomes in Breast Cancer Surgery Patients Across Surgical Modalities: A Prospective Study. Cancers.

[28] Tomita S., Yoshitake T., Matsunaga N., de Kerckhove M., Fujii M., Terao Y. (2024). Patient-reported outcomes and quality of life after breast-conserving surgery, mastectomy, and breast reconstruction assessed using the BREAST-Q questionnaire. Breast Cancer Res. Treat..

[29] van Zeelst L.J., Straten R., van Eekeren R.R.J.P., van Uden D.J.P., de Wilt J.H.W., Strobbe L.J.A. (2025). Chest wall perforator flap reconstruction in breast conserving surgery: Quality of life and limited complications in outpatient treatment. World J. Surg. Oncol..

